# Electrical alternans of the Q-T interval and fatal arrhythmias caused by neonatal cardiac tumor: a case report

**DOI:** 10.3389/fcvm.2025.1552916

**Published:** 2025-07-16

**Authors:** Ge Wang, Jin Ding, Hongguang Wu, Yanhong Chen, Xiaoqi Deng, Hairui Li, Haiying Li

**Affiliations:** ^1^ Department of Arrhythmia, Cardiovascular Medical Center, University of Hong Kong-Shenzhen Hospital, Shenzhen, China; ^2^ Department of Medicine, Shenzhen University, Shenzhen, China

**Keywords:** case report, neonatal cardiac tumor, electrical alternation, arrhythmia, sudden death

## Abstract

Primary cardiac tumors in the fetal and neonatal populations are rare. In neonates, these tumors may present with a range of clinical manifestations, from asymptomatic cases to severe arrhythmias, valvular obstruction, and cardiac dysfunction. This report describes a case of fatal malignant arrhythmia caused by a cardiac tumor in an 8-day-old neonate. The electrocardiogram at birth revealed multiple abnormalities, including ST-T segment alterations, intraventricular conduction block, and bundle branch block. By the sixth postnatal day, the patient developed rapid, polymorphic malignant ventricular arrhythmias. Despite aggressive treatment, the neonate ultimately suffered sudden cardiac death. This case underscores the potentially fatal risk of arrhythmias associated with cardiac tumors.

## Introduction

Cardiac tumors have been reported to significantly impair neonatal cardiac function, causing arrhythmias, valve malformations, and chamber obstruction, which can lead to sudden neonatal death (1–3). Arrhythmias examined in this study include Wolff-Parkinson-White syndrome (WPW), supraventricular tachycardia (SVT), ventricular tachycardia (VT), and atrioventricular block (AVB) (4, 5). The early onset of arrhythmias and electrical alternans may serve as indicators of heightened instability in cardiac electrical activity.

## Case report

**Figure 1:**
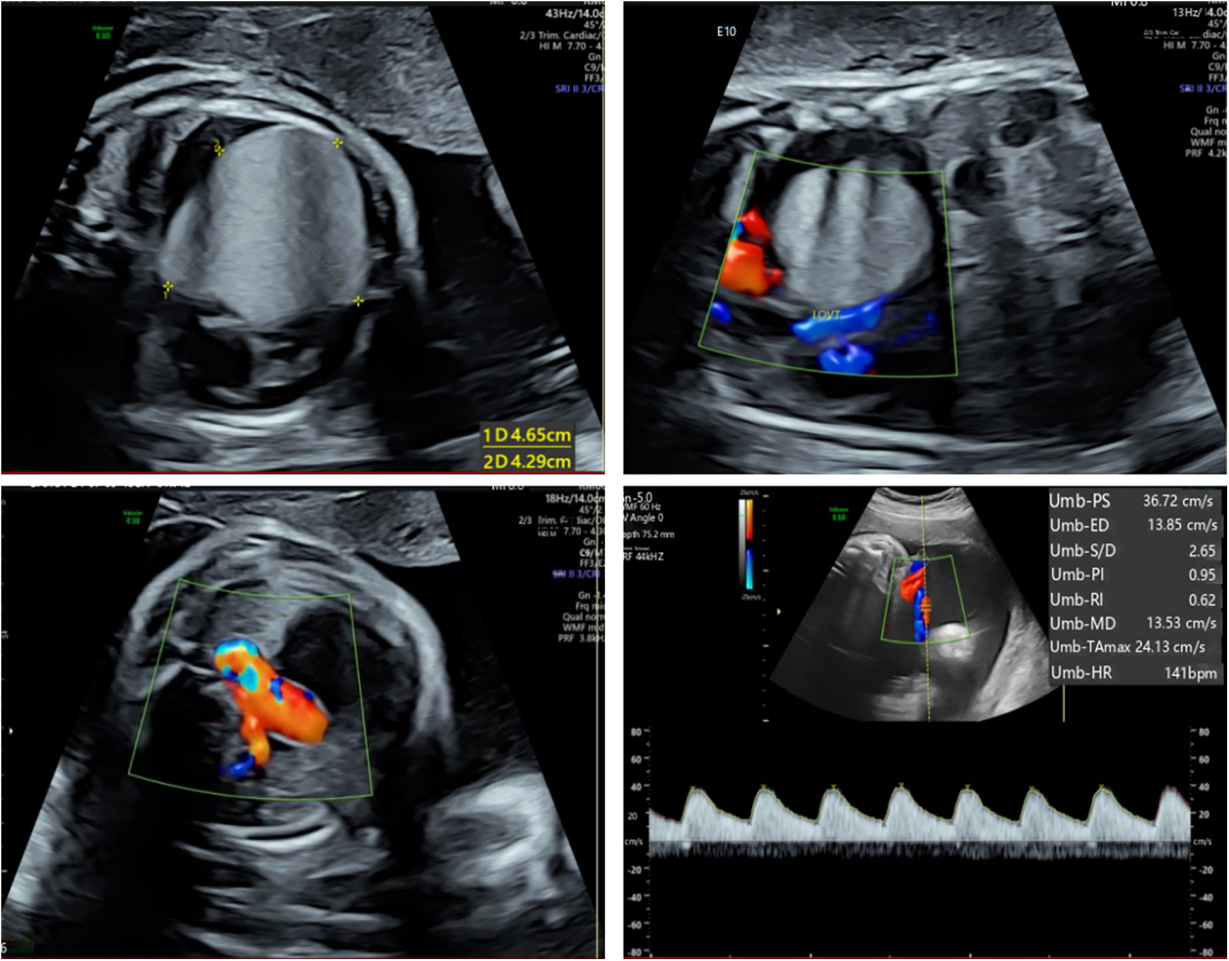
Fetal echocardiography at 34 weeks gestation. Fetal echocardiography detected a hyperechoic mass in the left ventricle, measuring approximately 47 × 44 × 40 mm.

The patient's mother was a 33-year-old Chinese woman who had not been exposed to any toxins, drugs, or other potential teratogenic substances during her pregnancy. Moreover, she had no family history of tumors. At 34 weeks' gestation, fetal echocardiography identified a hyperechoic mass in the left ventricle measuring approximately 47 × 44 × 40 mm (Figure 1). The mass exhibited uniformly distributed internal echogenicity and was well delineated from the surrounding myocardial tissue. Color Doppler ultrasound demonstrated an absence of blood flow within or around the mass. Notably, the mass extended partially into the left ventricular outflow tract, raising concerns about potential obstruction. Additional findings included mild tricuspid regurgitation, a slightly narrowed aortic arch, and a small pericardial effusion. Subsequent amniocentesis for fetal chromosomal karyotyping, chromosomal microarray analysis (CMA), and Trio whole-exome sequencing (Trio-WES) did not reveal any pathogenic variants.

The neonate was delivered via cesarean section at 38 weeks and 6 days due to potential complications associated with a cardiac mass. At birth, the male infant weighed 3,000 g and measured 50 cm in length. Apgar scores were 9 at one minute and 10 at both five and ten minutes. Vital signs were within normal limits, with a heart rate of 123 beats per minute, a respiratory rate of 42 breaths per minute, blood pressure of 62/46 mmHg, and a body temperature of 36°C. No initial signs of cardiac distress or other abnormalities were observed. Additionally, the neurological examination was unremarkable, and the initial electrocardiogram demonstrated a normal sinus rhythm.

**Figure 2:**
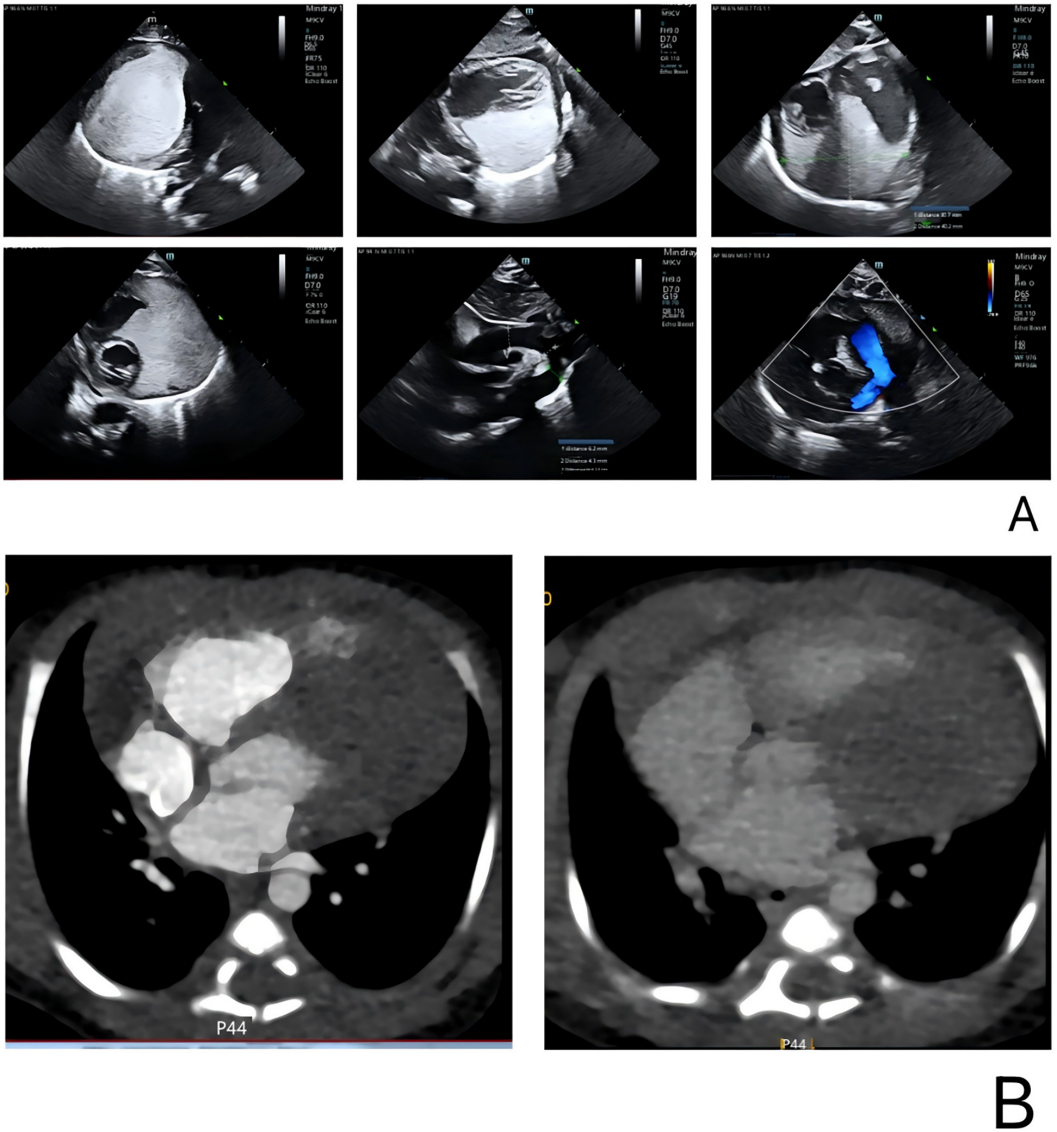
Cardiac color Doppler ultrasound and CT in the pediatric patient. **(A)** A cardiac ultrasound performed two days after birth revealed a hyperechoic region adjacent to both ventricles, as well as near the aorta and pulmonary artery. The lesion measured 40 × 34 × 36 mm, and no significant blood flow was detected on color Doppler imaging. **(B)** Cardiac enhanced CT showed a large irregular lesion in the pericardium, left side, clear edge, 62 × 33 mm, plain CT value 61 HU, enhanced scan showed mild homogeneous enhancement. Morphology of great vessels: Normal aorta, left ventricle, branches. Atria: Normal size left and right. Ventricles: Normal size left and right. Valves: Normal shape and density aortic, pulmonary, mitral, tricuspid.

This neonate was born without cardiac symptoms, and the initial neurologic examination and electrocardiogram were unremarkable. However, a follow-up cardiac ultrasound (Figure 2A) performed two days later revealed a hyperechoic mass situated between the myocardium and pericardium, adjacent to both ventricles as well as the aorta and pulmonary artery. The mass measured approximately 40 × 34 × 36 mm and exhibited a homogeneous internal echogenicity. There were no signs of compression on the interventricular septum or the left ventricular wall, and wall motion remained preserved. The differential diagnosis included rhabdomyoma and fibroma.

Cardiac computed tomography (CT) (Figure 2B) revealed a large, irregular mass located on the left side of the pericardium that was poorly demarcated from the aorta, pulmonary artery, and left ventricular wall, suggesting a neoplastic process. No abnormalities were detected in the coronary arteries or their branches; however, the aortic arch exhibited stenosis. CT scans of the head and abdomen did not demonstrate any significant findings. Additionally, electrolyte levels and troponin T were within normal limits. Cardiac color Doppler ultrasound was performed on the second day after birth, corroborating these observations.

Echocardiography and cardiac computed tomography confirmed patent blood flow within the left ventricle. Both *in utero* viability and normal postnatal troponin T levels were observed. Cardiac CT did not reveal significant coronary artery compression or ischemia. The abnormal electrocardiographic patterns were attributed to the tumor's impact on myocardial repolarization and depolarization. Given the tumor's size, the child commenced sirolimus chemotherapy post-birth. A biopsy was scheduled, necessitating a 48-hour discontinuation of sirolimus; consequently, after five days of treatment, sirolimus was paused in preparation for the procedure on the seventh day.

On the seventh day, the child screamed, and ECG showed an unrecognizable heart rhythm. Emergency measures, including cardiopulmonary resuscitation, brain protection, and tracheal intubation, were implemented. ECG revealed ventricular fibrillation and pulseless ventricular tachycardia. Despite repeated defibrillation, rhythm stabilization failed, reverting to pulseless ventricular tachycardia. During ongoing CPR, lidocaine, esmolol, amiodarone, and sodium bicarbonate were administered. The ECG waveform temporarily disappeared but was restored after intravenous epinephrine, though wide deformity ventricular tachycardia and torsade de pointes were present. Magnesium sulfate was also given. The neonate gradually returned to sinus rhythm with a heart rate of 90–100 beats per minute. Paroxysmal ventricular tachycardia, frequent ventricular premature beats, paired ventricular premature beats, and R-on-T phenomenon were noted.

On the eighth day, ventricular tachycardia recurred, leading to sudden hypotension, desaturation, multiorgan failure, anuria, systemic edema, and frequent malignant arrhythmia episodes. Balloon ventilation and epinephrine were used to maintain blood pressure, but the condition did not improve significantly, and polymorphic ventricular tachycardia persisted. The neonate experienced recurrent malignant arrhythmia, decreased blood oxygen and pressure, and was declared clinically dead after a flat electrocardiogram and unmeasurable blood pressure were diagnosed.

## Discussion

**Figure 3:**
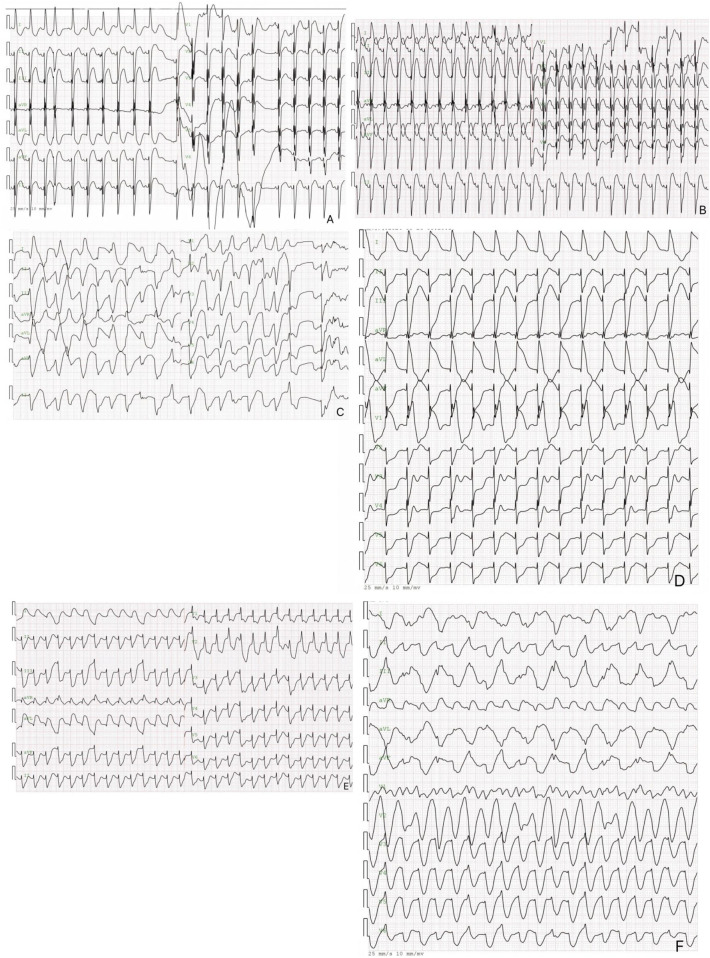
The electrocardiogram of the pediatric patient. 3 The image above is an electrocardiogram of a child soon after birth. The paper speed is 25 mm/s, 10 mm/MV. **(A)** Displays the results of the newborn's first ECG, showing sinus rhythm, nonspecific intraventricular block, ST segment elevation in inferior leads, and a prolonged Q-T interval. **(B)** Depicts the day 6 ECG results, which include sinus rhythm, left axis deviation, nonspecific intraventricular block, and ST-segment elevation. **(C)** Shows the day 7 electrocardiogram of the newborn, revealing multifocal/polymorphic ventricular tachycardia with intermittent torsade de pointes. **(D)** Presents the electrocardiogram results of the newborn on day 7, showing electrical alternans in the Q-T interval and left axis deviation. **(E,F)** depict a neonatal electrocardiogram on day 8, showing monomorphic ventricular tachycardia with ST-T segment changes.

**Figure 4:**
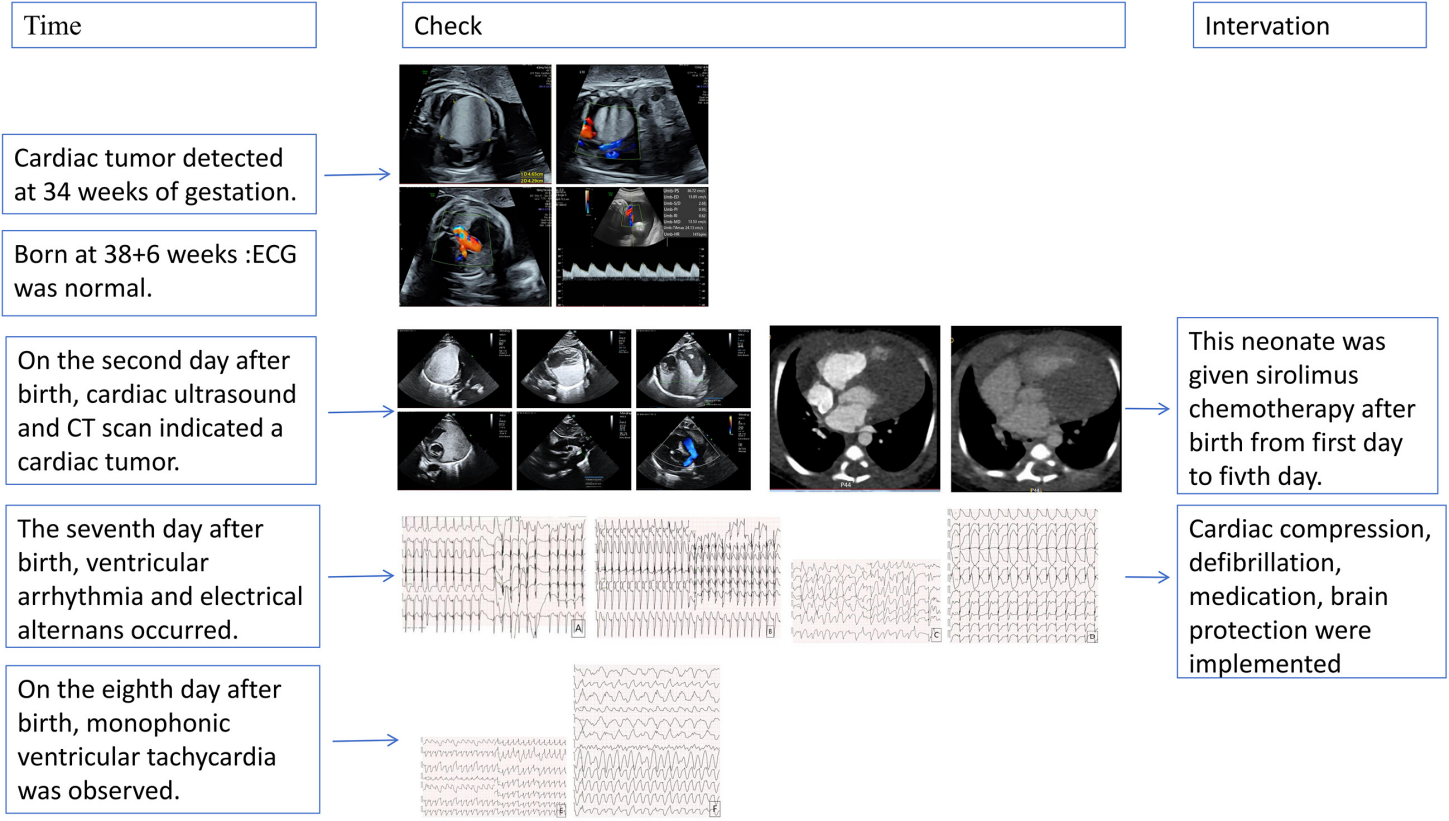
The timeline for the case's examinations and interventions.

This case illustrates the difficulties in treating neonatal ventricular tachycardia induced by a cardiac tumor, which caused severe arrhythmias unresponsive to pharmacologic and electrical treatments. Figure 3 shows the ECG results of the child measured from birth to the 8th day after birth. Although rare, neonatal cardiac tumors can significantly disrupt cardiac function and rhythm (6–8). The timeline for the case's examinations and interventions is shown in the Figure 4.

Sirolimus, also referred to as rapamycin, is a macrolide compound obtained from Streptomyces hygroscopicus. Its primary mechanism involves targeting mTORC1. Once it permeates the cell membrane, it binds to a cytosolic protein known as FKBP-12, thereby inhibiting mTORC1 activation. This inhibition disrupts cellular growth by halting the transition from the G1 phase to the S phase, ultimately contributing to the drug's beneficial impact on cell proliferation (9). While sirolimus does not target mTORC2 directly, extended treatment has been observed to diminish its activity. This reduction in mTORC2 function is linked to immunosuppressive effects on both T and B cells, as well as the development of glucose intolerance (10), possibly leading to new-onset diabetes mellitus (11). Rapalogs, in contrast, specifically target mTORC1, resulting in more beneficial metabolic effects (12). At present, there is a scarcity of comprehensive data on dosing, treatment duration, and safety profiles in newborns, with only a limited number of case reports available in the literature (13, 14). Nonetheless, these treatments appear promising for managing TSC symptoms and rhabdomyoma, with numerous reports indicating that they can significantly reduce the tumor's size (14).

At immunosuppressive dosages, both sirolimus and everolimus have been linked to various side effects. These include hematological issues (such as anemia, leukopenia, and thrombocytopenia), metabolic disturbances (including hypercholesterolemia and hypertriglyceridemia), diarrhea, among others. Furthermore, when sirolimus is combined with cyclosporine, there is evidence of elevated creatinine levels and diminished glomerular function. Notably, the study by Klawitter et al. demonstrated that these medications did not impair tubular, interstitial, or vascular functions (9). Nephrotoxicity was observed solely when sirolimus, administered at a dosage of 5 mg/kg/day, was combined with cyclosporin given at its maximum dose of 10 mg/kg/day (15). The condition may present as proteinuria or glomerulonephritis, particularly in the form of focal segmental glomerulosclerosis. Conversely, when paired with cyclosporin, everolimus does not appear to exhibit a synergistic effect that exacerbates nephrotoxicity (16). Other investigations examining pancreatic impairment have found no evidence to suggest a connection with the administration of either sirolimus or everolimus (17). In the case reported by us, due to the large cardiac tumor, sirolimus chemotherapy was given on the first day after birth. The drug concentration of simoros was 41.91 ng/ml from August 8 to 21, and sirolimus was discontinued on August 24, and a cardiac tumor biopsy was planned on August 26.

The neonate initially had a normal sinus rhythm and Q-T interval but developed ventricular tachycardia and torsade de pointes by day 7. Normal initial electrolyte and troponin T levels, along with a cardiac CT showing no coronary abnormalities, indicated that the arrhythmia was not due to electrolyte imbalances or coronary stenosis. Despite medical treatment and defibrillation, the newborn's electrocardiogram revealed Q-T alternans.

In our reported case, the family's decision to decline an autopsy prevented us from obtaining a pathological evaluation of the cardiac tumor. Fetal echocardiography, particularly when using the four-chamber view in the second trimester, has proven to be a highly sensitive method for detecting fetal cardiac tumors. Notably, C. Chen J et al. have documented several case series on this subject (18), Fetal cardiac rhabdomyomas were the most prevalent tumors and are typically small due to their self-limited growth. On fetal echocardiography, pathologically confirmed rhabdomyomas had maximum and median diameters of 23 mm and 11 mm, respectively. In contrast, both fibromas and hemangiomas reached maximum diameters of at least 30 mm, indicating a faster growth rate. Additionally, papillary fibroelastomas are commonly found attached on the downstream side of the valve, and the occurrence of multiple cardiac rhabdomyomas has been strongly associated with tuberous sclerosis complex (TSC) (19, 20). Fetal cardiac tumors have been reported to be associated with TSC at a rate of 30%–50% (21). Cardiac myxomas can vary significantly in size and are predominantly located in the left atrium. Nonetheless, the high distensibility and mobility of these tumors—especially when they are of the soft, myxoid type—can cause them to deform and become elongated during prolapse, thereby altering their apparent size (22). Rhabdomyomas are frequently observed as multiple lesions, commonly found in either the interventricular septum or the free wall of the heart (23). Conversely, fibromas generally present as solitary masses, most commonly found in the free wall of the left ventricle with infiltration into the myocardium, and they frequently undergo calcification (23). Rhabdomyomas, rhabdomyosarcomas, and leiomyosarcomas typically exhibit uniform echogenicity; nonetheless, central regions that appear brighter and more lucent may indicate the presence of necrotic areas or calcifications. In contrast, lipomas can present a spectrum of echogenic characteristics—from hypo- to hyperechoic—although they are generally consistent in appearance (24). Fibromas show usually an increased echogenicity compared to the normal myocardium (25–27). Hemangiomas, angiosarcomas, and lymphomas typically present with a heterogeneous echogenic profile, marked by scattered areas that appear echolucent (28–30). Sarcomas and lymphomas affecting either atrium can rapidly expand and cause obstruction by invading structures such as the mitral or tricuspid annulus. Similarly, pulmonary artery sarcomas are known to produce marked stenosis. In some instances, sarcomas extending from the atria may obstruct the venae cavae or pulmonary veins. Although rare, valvular fibroelastomas can result in valve regurgitation by exerting traction on the cusps. Additionally, large, solid extracardiac tumors may compress nearby vessels or cardiac chambers (24).

In this case, the patient's echocardiogram revealed a hyperechoic mass situated between the myocardium and pericardium. The mass was diffusely distributed and closely adhered to the left ventricle, right ventricle, and the regions corresponding to the main and pulmonary arteries, with dimensions of approximately 40 × 34 × 36 mm. Its internal echoes were relatively uniform, and the boundary with the myocardium was clearly defined. Cardiac computed tomography (CT) further demonstrated a large, irregular pericardial mass predominantly located on the left side, measuring about 62 × 33 mm. The non-contrast CT scan showed a value of approximately 61 Hounsfield units, while the contrast-enhanced scan revealed mild and uniform enhancement. Based on the echocardiographic and CT findings, a benign cardiac tumor was suspected; however, the absence of histopathological examination precluded a definitive diagnosis of the tumor type.

The rapid deterioration of the patient's condition shortly after birth presents significant challenges to clinicians. This case highlights the urgent need for optimized management strategies to prevent fatal arrhythmias in neonates with cardiac tumors. What proactive measures should be prioritized in the selection of treatment plans? The early occurrence of arrhythmias and electrical alternans may signal an increase in the instability of cardiac electrical activity, further prompting consideration of the feasibility and effectiveness of comprehensive treatment options such as partial resection of the cardiac tumor combined with implantation of an implantable cardioverter-defibrillator (ICD), extracorporeal membrane oxygenation (ECMO), and heart transplantation. In the cases we reported, combining echocardiography and enhanced cardiac CT, the patient's left ventricular blood flow was normal after birth. The tumor did not cause obstruction of the left ventricular inflow or outflow tracts, nor did it affect hemodynamics. There was no respiratory impairment or significant systemic embolism risk, and there were no indications for emergency surgery. Additionally, the cardiac tumor was located between the epicardium and myocardium, and the coronary artery runs through the heart surface, making surgical resection impossible. Chemotherapy combined with heart transplantation might be the only treatment option. To determine the nature of the tumor, a biopsy could be performed, but this would require the patient to stop using cyclosporine for 48 h. After the patient developed malignant arrhythmias, there was a significant disturbance in the internal environment, generalized edema, and recurrent malignant arrhythmias. The risk of biopsy is extremely high, as it may trigger ventricular fibrillation, and surgical procedures cannot be performed until the condition stabilizes. After the onset of cardiogenic shock, active anti-arrhythmic treatment and correction of internal environmental disturbances were initiated. However, the patient had gastrointestinal bleeding and neonatal necrotizing enterocolitis, and the family refused hemodialysis and surgical treatment, leading to the inability to correct generalized edema and electrolyte disturbances. Despite active anti-shock treatment, the patient's lactic acid levels showed a clear downward trend, but the recurrent malignant arrhythmias could not be prevented, ultimately resulting in the patient's death.

Studies suggest that electrical alternans might serve as a non-invasive test for electrophysiological instability (31). The neonate displayed a prolonged Q-T interval at birth, later developing ventricular tachycardia and fibrillation. Q-T interval alternans appeared as the disease progressed, but subsequent treatments failed to sustain sinus rhythm, implying that electrical alternans in humans may also indicate a reduced ventricular fibrillation threshold. This case emphasizes the malignant and lethal potential of tachyarrhythmias associated with neonatal cardiac tumors. Some experts suggest that effectively controlling ventricular tachycardia linked to benign cardiac tumors in infancy could lead to a good prognosis without future arrhythmias or drug treatment (32).

Electrical alternans (EA) refers to a phenomenon observed in electrocardiograms, where the amplitude and morphology of QRS complexes alternate between consecutive heartbeats. In the context of neonatal cardiac tumors—especially in cases linked to tuberous sclerosis complex (TSC) or involving multiple myocardial tumors—this alternation may signal underlying electrophysiological disturbances or damage to myocardial cells (33). Based on our reports, electrical alternans might serve as an early indicator of malignant arrhythmias, warranting additional ECG monitoring and thorough cardiac function evaluation. For example, one case involving a neonatal cardiac tumor presented with electrical alternans alongside ventricular tachycardia on the ECG, implying a potential arrhythmia risk (34). Accordingly, healthcare professionals should monitor electrical alternans closely and integrate its assessment into the ECG critical value protocols to promptly identify high-risk individuals and intervene appropriately.

Neonatal cardiac tumors are assessed according to various risk categories determined by factors such as size, anatomical placement, hemodynamic impact, and any association with genetic syndromes. For instance, low-risk tumors are typically small, do not hinder hemodynamic function, and are not accompanied by arrhythmias or heart failure. In contrast, medium-risk tumors are larger and may lead to slight hemodynamic impairment without eliciting clear symptoms. High-risk tumors tend to be substantial in size and can trigger significant hemodynamic disturbances, arrhythmias, or heart failure, which may necessitate surgical intervention (35, 36).

Conservative management: For tumors classified as low-risk, a strategy of careful observation and regular follow-up is typically preferred over immediate surgical intervention, as research has demonstrated that many cardiac rhabdomyomas tend to regress naturally after birth. Medication: In cases involving specific tumor types, such as multiple cardiomyomas linked to tuberous sclerosis complex, mTOR inhibitors like sirolimus can be administered to control tumor growth and alleviate arrhythmias (37, 38). Surgical treatment: For tumors deemed high-risk or intermediate-risk—particularly those that lead to hemodynamic instability or arrhythmias—surgical removal becomes a viable option. The decision regarding the timing and approach for surgery should be tailored to the tumor's location, size, and the patient's overall condition (39, 40). ICD: For patients susceptible to life-threatening arrhythmias, such as paroxysmal ventricular tachycardia or ventricular fibrillation, the implantation of an ICD should be contemplated as an interim measure to prevent sudden cardiac death (34, 41).

ECMO is an effective supportive technique used to treat patients with heart failure following neonatal cardiac surgery. Studies have shown that ECMO plays a crucial role in managing heart failure after cardiac surgery, especially when the patient cannot be taken off cardiopulmonary bypass (CPB) or when low cardiac output syndrome (LCOS) occurs (42, 43). A retrospective study found that 23 newborns who received ECMO support after cardiac surgery had a success rate of 78.26% in being weaned off ECMO and a discharge rate of 52.17%. However, the use of ECMO also carries a higher risk of complications, including bleeding, nerve damage, renal failure, and gastrointestinal bleeding (42). The surgical treatment of cardiac tumors depends on the tumor's location, size, and its impact on heart function. A case report describes a newborn with an intramyocardial tumor who underwent surgery on the 19th day after birth. The child recovered well within two months post-surgery, and no recurrence was observed during follow-up (42). Another newborn received surgery for a cardiac tumor and severe fetal edema, and the child recovered well post-surgery. Pathological examination revealed a mature cystic tumor (44). For benign cardiac tumors, the recurrence rate is low after complete resection, and common postoperative complications include heart failure, infection, and damage to adjacent structures or nerves. However, most patients ultimately survive. Recent studies have shown that ECMO can be used to treat complications associated with malignant tumors in certain cases. For example, a child with lymphoma suffered from cardiogenic shock due to tumor lysis syndrome and received ECMO combined with continuous renal replacement therapy (CRRT). This treatment successfully corrected the internal environment disorder and shortened the duration of ECMO support (45). In cardiac tumor surgery, the use of ECMO involves considering several factors, including the tumor's location, size, its impact on heart function, and the patient's overall condition. For instance, a newborn with a right atrial tumor received ECMO support before surgery to maintain circulatory function. Postoperative pathology revealed a cardiac hemangioma (46). Early identification and intervention of residual lesions is the key to improving postoperative prognosis. Studies have shown that early cardiac catheterization or CT angiography during ECMO support can significantly improve the rate of off-line and hospital discharge (47).

## Data Availability

The original contributions presented in the study are included in the article/Supplementary Material, further inquiries can be directed to the corresponding author.
