# Supplementary material for: Electrical alternans of the Q-T interval and fatal arrhythmias caused by neonatal cardiac tumor: a case report
Source: Front Cardiovasc Med. 2025 Jul 16;12:1552916. doi: 10.3389/fcvm.2025.1552916 (PMC12307388; doi:10.3389/fcvm.2025.1552916)
Supplement: Supplementary file 1 [file Table1.docx]

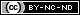

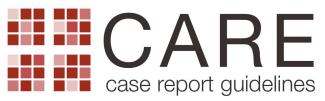

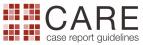

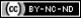

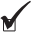
**CARE Checklist of information to include when writing a case report**


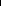


**Topic Item Checklist item description Reported on Line**

**Title**

**Key Words Abstract**

**(no references)**

**Introduction**

**Patient Information**

**Clinical Findings Timeline**

**Diagnostic Assessment**

**Therapeutic Intervention**

**Follow-up and Outcomes**

**Discussion**

**Patient Perspective Informed Consent**

**1** The diagnosis or intervention of primary focus followed by the words “case report” . . . . . . . . . . . . . . . . . . . . . . . . . . Title

**2** 2 to 5 key words that identify diagnoses or interventions in this case report, including "case report" . . . Key words

**3a** Introduction: What is unique about this case and what does it add to the scientific literature? . . . . . . . . . . . . . . . . Abstract

**3b** Main symptoms and/or important clinical findings . . . . . . . . . . . . . . . . . . . . . . . . . . . . . . . . . . . . . . . . . . . . . . . . . . . . . . . Abstract

**3c** The main diagnoses, therapeutic interventions, and outcomes . . . . . . . . . . . . . . . . . . . . . . . . . . . . . . . . . . . . . . . . . . . Abstract

**3d** Conclusion—What is the main “take-away” lesson(s) from this case? . . . . . . . . . . . . . . . . . . . . . . . . . . . . . . . . . . . . . Abstract

**4** One or two paragraphs summarizing why this case is unique (**may include references**) . . . . . . . . . . . . Introduction

**5a** De-identified patient specific information. . . . . . . . . . . . . . . . . . . . . . . . . . . . . . . . . . . . . . . . . . . . . . . . . . . . Case report

**5b** Primary concerns and symptoms of the patient. . . . . . . . . . . . . . . . . . . . . . . . . . . . . . . . . . . . . . . . . . . . . . . . . . . . . Case report

**5c** Medical, family, and psycho-social history including relevant genetic information . . . . . . . . . . . . . . . . . Case report

**5d** Relevant past interventions withoutcomes . . . . . . . . . . . . . . . . . . . . . . . . . . . . . . . . . . . . . . . . . . . . . . . . . . . . . . . . N/A

**6** Describe significant physical examination (PE) and important clinical findings. . . . . . . . . . . . . . . . . . . . . . . Case report

**7** Historical and current information from this episode of care organized as a timeline . . . . . . . . . . . . . . . Case report

**8a** Diagnostic testing (such as PE, laboratory testing, imaging, surveys). . . . . . . . . . . . . . . . . . . . . . . . . . . . . . . Case report

**8b** Diagnostic challenges (such as access to testing, financial, or cultural) . . . . . . . . . . . . . . . . . . . . . . . . . . . . . N/A

**8c** Diagnosis (including other diagnoses considered) . . . . . . . . . . . . . . . . . . . . . . . . . . . . . . . . . . . . . . . . . . . . . . . . . Case report

**8d** Prognosis (such as staging in oncology) where applicable . . . . . . . . . . . . . . . . . . . . . . . . . . . . . . . . . . . . . . . . . Case report

**9a** Types of therapeutic intervention (such as pharmacologic, surgical, preventive, self-care) . . . . . . . . . . . . . . . . . . . . Case report

**9b** Administration of therapeutic intervention (such as dosage, strength, duration) . . . . . . . . . . . . . . . . . . . . . . . . . . . . . Case report

**9c** Changes in therapeutic intervention (with rationale) . . . . . . . . . . . . . . . . . . . . . . . . . . . . . . . . . . . . . . . . . . . . . . . . . . . . Case report

**10a** Clinician and patient-assessed outcomes (if available) . . . . . . . . . . . . . . . . . . . . . . .. . . . . . . . . . . . . . . . . . . . . . . . . . . . N/A

**10b** Important follow-up diagnostic and other test results . . . . . . . . . . . . . . . . . . . . . . . . . . . . . . . . . . . . . . . . . . . . . . . . . . . . Case report

**10c** Intervention adherence and tolerability (How was this assessed?) . . . . . . . . . . . . . . . . . . . . . . . . . . . . . . . . . . . . . . . . . Case report

**10d** Adverse and unanticipated events . . . . . . . . . . . . . . . . . . . . . . . . . . . . . . . . . . . . . . . . . . . . . . . . . . . . . . . . . . . . . . . . . . . Case report

**11a** A scientific discussion of the strengths AND limitations associated with this case report . . . . . . . . . . . . . . . . . . . . . . . Discussion

**11b** Discussion of the relevant medical literature **with references**. . . . . . . . . . . . . . . . . . . . . . . . . . . . . . . . . . . . . . . . . . Discussion

**11c** The scientific rationale for any conclusions (including assessment of possible causes) . . . . . . . . . . . . . . . . . . . . . . . . Discussion

**11d** The primary “take-away” lessons of this case report (without references) in a one paragraph conclusion . . . . . . . Discussion

**12** The patient should share their perspective in one to two paragraphs on the treatment(s) they received . . . . . . . . N/A

**13** Did the patient give informed consent? Please provide if requested . . . . . . . . . . . . . . . . . . . . . . . . . . . . . . . . . . . . . . **Yes ☑ No ☐**
